# Molecular Epidemiology of *Epstein-Barr virus* (EBV) in Patients with Hematologic Malignancies

**DOI:** 10.31557/APJCP.2020.21.3.693

**Published:** 2020-03

**Authors:** Alireza Tabibzadeh, Mohammad Hadi Karbalaie Niya, Maryam Esghaei, Farah Bokharaei-Salim, Angila Ataei-Pirkooh, Seyed Jalal Kiani, Seyed Hamid Reza Monavari

**Affiliations:** 1 *Department of Virology, *; 2 *Gastrointestinal and Liver Disease Research Center, Iran University of Medical Sciences, Tehran, Iran. *

**Keywords:** Epstein-Barr virus (EBV), EBV Nuclear Antigen (EBNA), genotyping, hematologic malignancy

## Abstract

**Background:**

*Epstein-Barr virus* (EBV) is associated with different malignant diseases, such as Hodgkin lymphoma (HL) and lymphoproliferative disorders. Patients with hematologic malignancies by variable severity could be suspected for the infection with different types of this virus. This preliminary study reported the genotyping and related viral load of Epstein-Barr virus in Iranian patients with hematologic malignancies for estimation of possible factors affecting malignancy.

**Methods:**

Peripheral blood mononuclear cells (PBMC) of HL (n=20), NHL (n=29), acute lymphocytic leukemia (ALL) (n=18) and chronic lymphocytic leukemia (CLL) (n=12) were obtained. After DNA extraction, a nested-PCR and a conventional-PCR targeting *EBNA-2* and *EBNA-3C* genes were performed. A real-time PCR assay for viral load quantitation carried out. Standard curve analysis used for evaluation of amplification specificity.

**Results:**

Of 79 included patients, 34 (43%) were EBV positive. There were 23.5% (8/34), 38.2% (13/34), 23.5% (8/34), 14.8% (5/34) in HL, NHL, ALL and CLL groups, respectively. Also, the main genotype was genotype I (91.2%) which it follows by 8.8% (3/34) genotype II. The real-time PCR assay showed the mean viral load ± std. deviation was 2.75×10^5^ ± 1.202×10^6^ copies/μg DNA and the higher viral load was seen in NHL patients.

**Conclusion:**

This preliminary investigation in Iran shows that the main EBV genotype into our region probably is genotype I (91.2%) which it is similar to others. We could not find any statistically significant association between the virus infection and viral load with any specific disease and patients’ demographic data.

## Introduction

Epstein-Barr virus (EBV) is a lymphotropic virus that is a member of one of the greatest virus families the Herpesviridae, and categorize in the gamma herpes virus genus. It has a linear, double-stranded DNA genome by approximately 170-kilobases in length. It was discovered and introduced by Epstein et al., (1964) from a Burkitt lymphoma cell line for first time. Also, EBV was the first human virus presumed to be oncogenic (Virus et al., 1997). Investigations estimated that about 90% of the adult population worldwide is infected by this virus. In developing regions, primary infection by EBV mostly occurs in a few years after birth which it is asymptomatic. While this primary infection occurs in adolescence or adults it can cause self-limiting infectious mononucleosis syndrome (Williams and Crawford, 2006). Viral infection occurs through saliva which it leads to productive oropharyngeal infection. For maintaining lifelong persistence infection, latent infection in circulating B cells triggered by episomal forms of the viral genome in B cells nuclei (Gandhi et al., 2004; Küppers, 2003; Young and Rickinson, 2004). EBV latency is accompanied by the low transcription of its nuclear antigens (EBNA), latent membrane proteins, small noncoding RNAs, and BamHI-A transcripts (Ambinder and Weiss, 1999). EBV can run latency in different strategies depends on the differentiation of B cells. These strategies cause higher survival of EBV-infected cells and viral immune evasions from adaptive immune responses (Chen et al., 2006; Rezk and Weiss, 2007; Thorley-Lawson, 2001). 

EBV infection is associated with a wide range of malignant and non-malignant diseases. It has been linked to many human neoplasms, for instance, Hodgkin lymphoma (HL), Burkitt lymphoma (BL), lymphoproliferative disorders in immunocompromised individuals (PTLD), nasopharyngeal carcinoma, gastric carcinomas, inflammatory pseudo-tumors of the liver and spleen, HIV-associated smooth muscle neoplasms, and EBV-associated lymphoproliferative disorders (Arber et al., 1995; Iezzoni et al., 1995; Lee et al., 1995; Rezk and Weiss, 2007; Yamamoto et al., 1994).

Studies indicated that EBV genetic variants and geographical regions could have a key role in EBV-associated diseases, but the exact relation is still on debate. By considering nuclear antigen sequences (EBNA-2, -3A, -3B, and -3C), EBV can divide in type I and II (Adldinger et al., 1985; Sample et al., 1990). Although, studies on Iranian HL and NHL patients showed the EBV and other herpesviruses such as Kaposi’s sarcoma-associated herpesvirus (KSHV) frequency; there were some controversies in the association of EBV infection and its genotypes in these patients (Hesamizadeh et al., 2016; Keyvani et al., 2017). In this preliminary study in Iran, we aimed to detect the presence of EBV genome, EBV genotyping and then viral load quantitation based on EBNA-2 and EBNA-3C genes via molecular techniques, in patients with different hematologic malignancies and chronic lymphoproliferative disorders to introduce their potential risk factors with exposure to EBV infection.

## Materials and Methods

*Patient selection*

Specimens were obtained from patients with hematologic malignancies included HL, non-Hodgkin’s lymphoma (NHL), acute lymphoblastic leukemia (ALL), chronic lymphocytic leukemia (CLL) that diagnosed by sophisticated hemato-oncologist based on clinical manifestations and laboratory findings, enrolled into the current cross-sectional study. Patients were conducted by hospitals affiliated with the Iran University of Medical Sciences, Tehran, Iran between Dec 2016 and Feb 2018. The inclusion criteria were the positive diagnosis of one of the mentioned hematologic malignancies (established on clinical evaluation included lymph node biopsy and flow cytometry on peripheral blood, bone marrow aspirate, and so on) and written informed consent and the exclusion criteria includes, patients with other hematologic disorders, no established diagnosis patients and non-availability of the written informed consent. All procedures were reviewed and approved by the Committee of Human Studies at the Ethics Committee of Iran University of Medical Sciences, Tehran, Iran by the record number IR.IUMS.REC.1396.32501.

*Sample preparation*

Peripheral blood samples were collected as 5 cc from each patient into EDTA-containing tubes. Hematological parameters [white blood cells (WBCs) count, hemoglobin (Hochberg et al., 2016) test and platelet (Plt) count] were analyzed by the Sysmex XE-2100 (Sysmex Corporation, Kobe, Japan), and the PBMCs were collected from the whole blood by standard method under centrifugation over a density gradient (Lymphoprep, Oslo, Norway) (Hesamizadeh et al., 2016). DNA was extracted from the PBMCs pellet by using a high pure viral nucleic acid kit (Roche Diagnostics GmbH, Mannheim, Germany) according to the manufacturer’s protocol. Evaluation of purified nucleic acids was done by NanoDrop ND-1000^® ^(Thermo Fisher Scientific Inc., Waltham, MA, USA) spectrophotometry. Isolated DNA was kept at -20˚C before use. EBV positive cell line was got from the national cell bank of Iran (Pasture Institute of Iran, Tehran, Iran) that extracted by QIAamp Fast DNA Tissue Kit (QIAGEN, Hilden, Germany) according to manufacturer instructions.

*EBV detection*

A nested-PCR (nPCR) performed for EBV infection detection via *EBNA-2* gene. The first round forward primer 5’-GCG GGT GGA GGG AAA GG-3’ and reverse 5’-GTC AGC CAA GGG ACG CG-3’and the second round forward 5’-AGG CTG CCC ACC CTG AGG AT-3’ and reverse 5’-GCC ACC TGG CAG CCC TAA AG-3’ were used by 240 and 170 bp amplicon sizes, respectively. For positive control we used Raji (EBV+) cell line extract. The reaction mix contains 0.2–0.5 µM of template DNA or controls, 0.5 mM of dNTP mix (Fermentas GmbH, Germany), 0.5 µM of each primers, 1.5 µM MgCl_2 _(Fermentas GmbH, Germany), 5 units/µl of Taq DNA polymerase (Fermentas GmbH, Germany) and sterilized DW added to round out the total volume to 25 µl for each reaction. A Bio-Rad thermocycler (T100™ Thermal Cycler) was used for heating programs. Thermocycler heating protocol was included 5 min at 95^o^C, 40 cycles of the 30s at 95^o^C, 30s at 56^o^C (for the first round) and 60^o^C (for the second round), 40s at 72^o^C and one final extension step at 72^o^C for 5 min. For visualization of PCR products via UV transilluminator, a gel electrophoresis was performed by Tris-Boric Acid-EDTA 1× (TBE) buffer system and the 1.5% agarose gel stained by Ethidium bromide.

*EBV genotyping*

A conventional-PCR (cPCR) performed for detection of EBV genotypes by *EBNA-3C* gene. A set of EBNA-3C primers was used included forward 5’-CGG AAG AGG TGG AAA ACA AA-3’ and reverse 5’-GTG GGG GTC GTC ATC ATC TC-3’. This primer set could differ EBV type I and II by different PCR product sizes 153 and 246 bp, respectively. For positive control we used Raji cell line extracts. PCR conditions included master mix preparation and the heating protocol was as nPCR except 58^o^C for the annealing step. 

*Quantitative real-time PCR*

A SYBER Green quantitative real-time PCR (qRT-PCR) performed for viral load quantitation via EBNA-3C specific primers. Reaction mixture contains 7.5µl SYBR RT-PCR Master Mix (Odense M, Denmark) corresponds to 1X concentration in each tube, 0.5 µl of each mentioned forward and reverse primers (10 pmol/µl), 0.2–0.5 μM concentration of each sample or controls, distilled water added for the rest of the 15 µl total volume. The Rotor-Gene-Q 6000 thermocycler (Corbett, Australia) was used for Real-time PCR. The heating program was a preheating step 5min at 95^o^C, 40 cycles of 95^o^C for the 30s and 60^o^C for 30s. The reaction specificity was achieved by melting curve analysis. The melting curve was adjusted for 50 to 99^o^C by 5s Intervals. A standard curve efficiency 0.95 to 1.05 used by a serial dilution manner of standards. Generay Biotechnology (Generay Biotechnology Co, Shanghai, China) performed for cloning the primers zone into pTZ57R/T plasmid. To improve the accuracy of the analysis, standards were duplicated. 

*Statistical analysis*

For the statistical analyses in this study we used the SPSS version 22 software (SPSS Inc., Chicago, IL, USA). Based on the variables we assessed the statistically significant with the Chi-square and Mann–Whitney U test. The statistically significant were considered as p-value<0.05.

## Results

**Figure 1:**
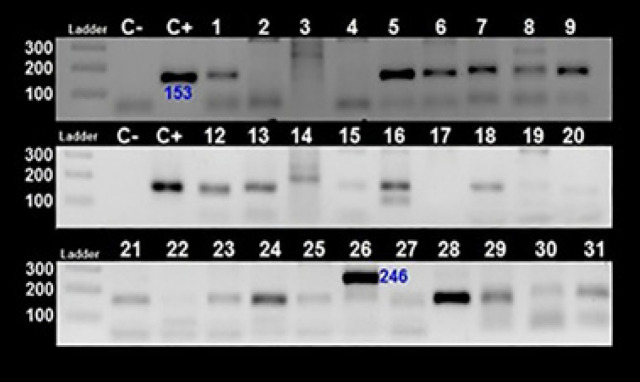
EBNA-2 nPCR for Detection of EBV Infection. Top, first round (product length 240 bp) and bottom, second round (product length 170 bp) of nPCR. C-, NTC; C+, Raji (EBV+) cell line extract

From a total of 79 included patients by the mean age (y) ± std. deviation 57.7±18.8, 44 (55.7%) were male. Included patients were composed of 19 HL, 28 NHL, 20 ALL and 11 CLL subjects. Details of demographic features of included patients showed in table 1. Based on EBNA-2 nPCR results we found 43% (34/79) EBV positive that the majority of them were male (61.8%). Figure 1 showed results of some EBV positives round 1 and 2 nPCR. 

Of the 34 EBV positive strains, 26.5 % (9/34) were HL, 38.2% (13/34) were NHL, 26.5% (9/34) ALL, 11.8% (4/34) were CLL. Cell blood count (CBC) of WBCs, Hb and Plt for each groups showed there were not any differences in each group. Details are summarized in Table 1. 

**Figure 2:**
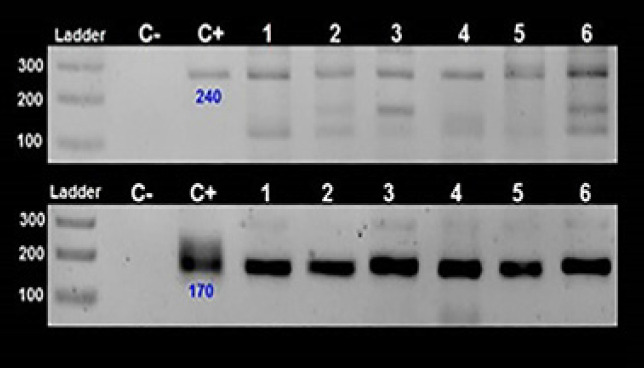
EBV Genotyping by EBNA-3C Specific Primers. EBV genotype 1 and 2 have a 153 and 246 bp band sizes, respectively. No 8 and 26 are genotype 2 and others are genotype 1. No 2, 3, 4, 14, 17 were negative or has non-specific bands. C-: NTC; C+: Raji (EBV+) cell line extract

EBV genotyping (based on different amplicon sizes) by EBNA-3C specific primers showed the majority of EBV positive strains were genotype I (91.2%) and 8.8% (3/34) were genotype II. Figure 2 showed some cPCR for *EBNA-3C* gene results. 66.7% (2/3) EBV-II were found in HL and 33.3% (1/3) was in NHL patients. 

A qRT-PCR performed for viral load quantitation via serial dilution manner of the cloned standard into pTZ57R/T plasmid. After evaluation of primers specificity by melting curve analysis, a standard curve (E=1.06) used for EBV positive strains analysis. The mean ± std. Deviation of viral loads were 2.75×10^5^ ± 1.202×10^6^ copies/μg DNA that the higher viral load was in NHL patients but the results could not shows any statistical significance difference between the groups (p>0.05). Details are summarized in Table1.

Statistically, there were not any significant differences between EBV infectivity and the four groups of lymphoproliferative diseases and chronic blood disorders. Statistics showed there were not any significant association between age (p=0.1), gender (p=0.3), and EBV positivity. Analysis of different variables (age, gender, WBC, Hb and Plt) in each four groups (HL, NHL, ALL, CLL) with EBV positivity, genotype and viral load had not any significant result (p>0.05). Regardless of statistic relations, this data has shown that there was no type two EBV in ALL and CLL patients. Also, the majority of EBV positivity was seen in NHL although more EBV type 2 was seen in HL patients. 

**Table 1:** Demographic Characteristics of Patients with Lymphoproliferative Disorders (N=79)

	Parameters	HL n =20	NHL n =29	ALL n =18	CLL n =12	Total n =79
Total n=79	mean age^£^ ± SD^¥^	54.0±18.5	59.3±18.8	53.7±17.9	63.5±18.8	57.7±18.8
	Male No. (%)	13 (65)	14 (48.2)	11 (61)	6 (50)	44 (55.7)
	mean age ± SD	58.1±15.1	59.0±16.8	54.2±20.0	56.8±28.6	57.5±18.3
	Female No. (%)	7 (35)	15 (51.7)	7 (39)	6 (50)	35 (44.3)
	mean age ± SD	45.0±23.2	60.2±21.7	56.7±17.7	69.1±6.4	58.0±19.6
	Hb^ȵ^	11.2±1.2	11.1±1.1	10.9±1.3	11.0±1.3	11.1±1.2
	WBC^∞^	13.7±2.8	14.1±3.1	13.9±3.1	13.5±3.0	13.9±3.0
	Plt^π^	213.1±21.8	216.5±24.3	207.2±30.7	213.3±26.3	236.1±210.4
EBV Positive n=34 (43%)	No. (%)	8 (40.0)	13 (44.8)	8 (44.4) 52.4±21.1	5 (41.6)	34 (43.0)
	mean age ± SD	61.1±14.7	59.8±22.5		59.7±33.6	57.8±21.1
	Male No. (%)	7 (87.5)	5 (38.5)	6 (75)	3 (60)	21 (61.8)
	mean age ± SD	64.5±11.4	65.8±12.1	51.8±24.0	45.0±49.4	58.4±20.2
	Female No. (%)	1 (12.5)	8 (61.5)	2 (25)	2 (40)	13 (38.2)
	mean age ± SD	34	56.1±27.2	54.5±9.1	74.5±9.1	57.0±23.3
	Hb	10.6±1.5	10.7±1.1	10.9±1.5	10.2±0.8	10.7±1.2
	WBC	13.3±3.1	14.8±3.3	12.9±2.8	10.9±0.7	13.4±3.1
	Plt	223±25.0	215±27.9	204±42.4	192±11.2	212±30.8
Viral load€	mean ± SD	2.88e5 ± 8.391e5	5.15e5 ± 1.835e6	2781 ± 2.885e3	6.38e5 ± 1.259e6	2.75e5 ± 1.202e6
	Range	577 - 2526043	280 - 6622998	280 - 9750	1740 – 2526043	280 - 6622998
EBV Genotype I/II		6/2	12/1	8/0	5/0	31/3

^£^, years; ^¥^, Standard deviation; ^ȵ,^, Haemoglobin (g/dL); ^∞^, white blood cell (10^3^/µL); ^π^, platelet (10^3^/µL); ^€,^ copy per μg DNA

## Discussion

EBV as an oncogenic virus could be presence in different stages of B-cell maturation, and could infect certain epithelial cells. EBV pathogenic consequences could establish various lymphomas and carcinomas that its presence in the malignant cells could use for specific viral therapy in addition to cancer therapy (Pattle and Farrell, 2006).

In the present study, we aimed to find an association between EBV infection and hematologic malignancies as a preliminary molecular study in Iranian population. The probable roles of the viruses in development and pathogenesis of EBV in lymphoproliferative disorders and hematologic malignancies were quietly studied (Haque et al., 2002; Heslop, 2005; Park et al., 2007; Uner et al., 2011; Weiss et al., 1991; Weiss et al., 1987). In the present study, patients with hematologic malignancies were analyzed for EBV infection. Among 79 patients which grouped into HL, NHL, ALL and, CLL groups, EBV infection detected 40% (8/20), 44.8% (13/29), 44.4% (8/18), and 41.7% (5/12), respectively (p-values were not significant). The presence of EBV genome into these patients PBMCs showed a probable potential of EBV in their complications although there was not any antibody data of specific IgM and IgG for better EBV comprehensions. 

EBV based on encoded its nuclear antigens (EBNAs) divided into two genotypes I and II. Investigations on different EBV genotypes shows that the EBV-I can promote transformation better than EBV-II in human B cell lines (Rickinson et al., 1987). This data suggested there is a possible relation between virus genotype and inducted disease by considering their geographical and epidemiological patterns. Our results showed the majority of EBV-II were in HL patients since it proposed to more complications than others. As it has been indicated in studies the type I is distributed all around worldwide while the type II is mostly limited to the Equatorial Africa and Southeast Asia (Chang et al., 2009; Tzellos and Farrell, 2012). Investigations on Asian population shows that they mostly infected with one type which it was EBV-I and only 11% of them were co-infected with both types I and II (Srivastava et al., 2000; Tiwawech et al., 2008; Zhou et al., 2001). However, Porakbary et al., (pourakbari et al.) by used EBNA-2 cPCR for EBV detection in Iranian HL and NHL patients that they reported EBV prevalence 41.6% and 33%, respectively. There is limited data about different types of EBV in Iranian population with hematologic malignancies. In the study conducted by Mahjor et al., (2010) the EBV IgG antibody against early antigen (EA) and EBNA-1 were assessed in ALL patients in compare with control group. The study results indicated higher positive results for these antibodies by ELISA in ALL group. Also, in the study conducted by Ashraf et al., (2012) presence of EBV, LMP gene were investigated in diffuse large B cell (DLBL) patients by Immune-Histo-Chemical (IHC) and PCR. The results reveal 10% prevalence of LMP by PCR in DLBL patients. Furthermore, Habibian et al., (2013) showed the *EBV, EBNA-1 *gene presence in 48% of NHL patients by nested-PCR. Mean while, Jadali et al., (2008) indicates that 69.4% of the pediatric patients with HL had EBV positive results by using In situ hybridization (ISH) for *EBER* gene and IHC for *LMP-1* gene in lymph nodes tissue. Lin et al., (1993) investigated the prevalence and genotypes distribution of EBV in HL patients that they reported 56% of them were infected with type I and 13% were type II. Also, they found 31% co-infection by both genotypes. Ai et al., (2012) studied Chinese children for EBV infection that they reported 98.1% EBV type I vs. 1.9% type II. Our result showed there was not any co-infection of both EBV types that it could be related to geographical differences and Iranian race or our study specific group and limited sample size although there was not a confirmatory test to clarify some of our probable type II or mixed forms. Further studies in different groups and broader sample size needed to better clarify the subject.

Investigations showed there are different approaches for EBV genotyping and differentiate this virus variants. EBNA-3A, -3B, 3C and EBNA-2 were used for EBV genotyping but the most studied and approved the method for determining virus into the two distinct genotypes is to use EBNA-3C. Regardless of differentiating virus based on EBNA genes into two distinct genotypes, viral variant classification by EBNA-1 or different regions of the* LMP-1* gene sequence (Edwards et al., 1999; Snudden et al., 1995). By considering all this dividing the virus into two different variants based on EBNA-3C is still the most common method which is also clearly able to make difference between pathogenesis and geographical patterns of the virus while there is not any clear result about other variants of the virus yet. 

In the present study EBV infection in our population was detected by EBNA-2 nPCR and then EBV positive strains divided into two groups of genotypes I or II by EBNA-3C cPCR, and EBV viral load performed by a qRT-PCR. Based on the collected data we showed that the majority of EBV positive strains were genotype I (91.2%) and 8.8% (3/34) were genotype II. There is no clear cause for EBV geographic variations in different regions but this could be related to host HLA polymorphisms and environmental factors (Chang et al., 2009; Chiara et al., 2016). The needs to further genotype distribution studies to clarify their epidemiology and complications are sensible.

EBV viral load could be important for pathogenesis and disease prognosis especially in patients with malignancy. Studies on EBV viral load and its relation to different conditions showed the promising result. Smets et al., (2002) indicated that primary infection of EBV in PTLD (Posttransplant lymphoproliferative disease) patients induce high viral load and decrease T cell response to EBV. They concluded that the high viral load could indicate the risk of PTLD in patients. By the present study we determined the viral load of EBV in Lymphoproliferative disorders and hematologic malignancy patients via a qRT-PCR assay for EBNA-3C gene but limited data caused missing the status of T-cell in the studied population. Although we could not find significant results between viral load and different diseases or other demographic data (p>0.05), the highest viral load were seen in NHL patients that could be due to the broader sample size or their more severe malignancy compare with other studied patients. But it should considered that, this higher viral load in NHL patients were not statistically significance with other patients groups (p>0.05). The mean viral loads in our tested samples were 2.75×10^5 ^± 1.202×10^6^ copies/μg DNA. A major limitation of this study was to the small sample size that it was due to the special group of our study setting by inclusion criteria. Another limitation was the lack of using antibody data of EBV specific IgM and IgG. But our study for the first time in Iran showed the molecular epidemiology of the EBV in our special population groups and there was no study about EBV genotyping and viral load quantitation in Iranian population that it needs to confirm by complementary further studies.

In conclusion, by the current study, we were not found statistically significant data for association of any particular hematologic malignancies and different EBV genotypes or viral load. We concluded nearly half (43%) of our population had EBV genome and 91.2% of them were EBV I. The mean viral load was not determine any particular pattern with the disease and patients conditions. Further studies recommended for more comprehensive results.
